# Appearance and Frequency of Deep Venous Thrombosis After Total Hip Arthroplasty

**DOI:** 10.14789/jmj.JMJ21-0056-OA

**Published:** 2022-09-09

**Authors:** KOJI NAMIKI, MASAHIKO NOZAWA, SUNG-GON KIM, YUKO SAKAMOTO, HIRONORI OCHI, SUGURU KATO, MUNEAKI ISHIJIMA

**Affiliations:** 1 Department of Orthopedics, Juntendo University Graduate School of Medicine, Tokyo, Japan; 1 Department of Orthopedics, Juntendo University Graduate School of Medicine, Tokyo, Japan; 2 Department of Orthopedics, Juntendo University Nerima Hospital, Tokyo, Japan; 2 Department of Orthopedics, Juntendo University Nerima Hospital, Tokyo, Japan; 3 Department of Orthopedics, Juntendo University Juntendo Hospital, Tokyo, Japan; 3 Department of Orthopedics, Juntendo University Juntendo Hospital, Tokyo, Japan

**Keywords:** deep venous thrombosis (DVT), pulmonary embolism (PE), ultrasonography, total hip arthroplasty (THA)

## Abstract

**Objectives:**

Postoperative venous thromboembolism is an important peri-operative complication associated with total hip arthroplasty (THA). In particular, early detection of deep venous thrombosis (DVT) is important for the prevention of pulmonary embolism (PE). However, the methods and timing of examinations for DVT detection differ among the facilities. This study aimed to clarify the time, site, and frequency of DVT after THA.

**Materials and Methods:**

Background characteristics including age, sex, body mass index, diagnosis, operation type, operation time, and anesthesia type were investigated in 348 patients who underwent THA at our hospital between April 2017 and April 2019. Blood tests were performed preoperatively and on postoperative days (POD) 0, 1, 3, 7, 14, and 21. Simultaneously, vascular ultrasonography was performed to investigate the time and site of thrombus occurrence before and after the surgery.

**Results:**

DVT was observed in 27.2% of the patients on POD 21. The DVT-positive ratio was 9.4% (6/64) in males and 31.3% (89/284) in females. There was a significant difference between the groups (*p* = 0.0002). Patients in the DVT-positive group were significantly older than those in the DVT-negative group (73.0 ± 7.9 years vs. 63.3 ± 11.2 years, *p* = 0.0041). DVT mainly occurred in the soleal vein (74.7%). However, there was no significant difference between the operated and non-operated sides. In the DVT-positive group, thrombus occurred in 13.3% of preoperative cases, 20.0% on POD 0, 46.7% on POD 1, 13.3% on POD 3, 6.7% on POD 7, and 0% on POD 14 and 21.

**Conclusions:**

Vascular ultrasonography showed that thrombus occurred most frequently in the soleal vein. Thrombus occurred in 66.6% of DVT-positive patients by POD 1, indicating that thrombus appeared very early after surgery. All thrombus cases were formed by POD 7.

## Introduction

Owing to the current trend in aging societies, the number of total hip arthroplasty (THA) operations will increase in the future. THA is associated with various peri-operative complications, among which postoperative venous thromboembolism (VTE) is particularly important.

VTE combines deep venous thrombosis (DVT) and pulmonary embolism (PE). DVT is caused by thrombus formation in deep veins such as the iliac, femoral, popliteal, and lower leg veins. Thrombus release from the deep veins can be considered a cause of PE. After release, the thrombus flows into the bloodstream, passes through the right atrium and ventricle, and embolizes the pulmonary artery.

The frequency of DVT diagnosed by venography without prophylaxis in patients who have undergone THA was estimated to be 40-60% in the 7^th^ American College of Chest Physicians (ACCP) Guidelines^1)^ and 30-50% in the Japanese prevention guidelines^2)^. According to the 7^th^ ACCP Guidelines, postoperative symptomatic PE and fatal PE occurred in 1-30% and 0.1-7.5% of cases, respectively^1)^, compared to approximately 1% and 0.1% of cases, in the Japanese prevention guidelines^2)^.

The early detection of DVT is important for the prevention of PE because PE can lead to fatal complications. However, the methods and timing of examinations for DVT detection differ among the facilities. This study aimed to investigate and clarify the time, site, and frequency of DVT occurrences after THA.

## Materials and Methods

Written informed consent was obtained from all participants, and the study protocol was approved by the appropriate ethics committee (approval number: 17-37). Background characteristics such as age, sex, body mass index (BMI), diagnosis, type of operation, operation time, and type of anesthesia were investigated in 383 patients who underwent THA at the Juntendo Nerima Hospital between April 2017 and April 2019. Patients with rheumatism, use of preoperative anticoagulants, female hormones or steroids, and a history of VTE were subsequently excluded.

Table 1 shows the inspection flowchart. Blood tests of the coagulation system, such as D-dimer and fibrinogen degradation products (FDP), and vascular ultrasonography (US) of the lower limbs were performed preoperatively and on postoperative days (POD) 0, 1, 3, 7, 14, and 21 to investigate the time and site of thrombus occurrence before and after surgery.

**Table 1:** Flow chart of patient background surveys and blood sampling/ultrasound examinations

Perioperative	Pre	POD 0	POD 1	POD 3	POD 7	POD 14	POD 21
Patient background	●						
Blood Test	●	●	●	●	●	●	●
Contrast enhanced CT or pulmonary perfusion scintigraphy					●		
Ultrasonography	●	●	●	●	●	●	●

POD, post-operative day

The US of the lower extremities was performed using a whole-leg US method, and we were able to observe the femoral and popliteal veins continuously using a compression method. Subsequently, the posterior tibial, peroneal, soleal, and sural veins were carefully observed. Visualizing the veins with a probe, we defined cases in which there was no obstruction of the lumen of the vein by compression of the probe and the blood flow signal was missing in the color Doppler as a positive finding. Furthermore, we classified patients as DVT-positive if they had at least one DVT positive finding by US between preoperative and POD 21, and DVT-negative if they had no DVT positive findings. The presence of PE was examined using contrast-enhanced computed tomography (CT) or pulmonary perfusion scintigraphy on POD 7 for all patients.

Continuous variables are summarized as means (standard deviation). Normally distributed continuous data sets were analyzed using Student’s two-sample t-test. Categorical data was analyzed using Fisher’s exact test. All the tests were two-sided, and a *p*-value < 0.05 was considered statistically significant. Statistical analysis was performed using GraphPad PRISM Version 7.03 (GraphPad Software, 2365 Northside Dr. Suite 560, San Diego, CA 92108, USA).

## Results

We examined the occurrence of DVT using whole-leg US after THA in 383 patients from April 2017 to April 2019. For the present study, 35 patients were excluded because of rheumatics (n = 4), use of preoperative anticoagulants (n = 23), use of steroids (n = 3), a history of thrombosis (n = 5), and none of the patients used female hormones.

The remaining patients comprised 64 men with a mean age of 53.5 ± 10.4 years (range, 39-73 years) and 284 women with a mean age of 67.5 years (range, 48-90 years). The diagnoses were osteoarthritis in 330 cases and osteonecrosis (non-steroid) in 18 cases. The mean operation time was 130.1 ± 34.9 min (range, 115-158 min). All surgeries were performed under general anesthesia. Cementless implants were used in all patients (Table 2).

**Table 2:** Demographic and surgical characteristics of the patients

	Male	Female	Over-all
No. of patients	64(18.4%)	284(81.6%)	348
Age(years)	53.5±10.4(39-73)	67.5±10.4(48-90)	66.0±11.2
BMI (%)	25.7±2.1(21.8-32.8)	22.3±4.2(17.6-24.7)	22.8±2.75
Diagnosis			
Osteoarthritis	52	278	330(94.8%)
Osteonecrosis	12	6	18(5.2%)
Others	0	0	0
Type of operation			
Primary	64	284	348
Revision	0	0	0
Operation time(min)	132(115-153)	129.9(93-158)	130.1±34.9
Type of anesthesia			
General	64	284	348
Spinal	0	0	0

BMI, body mass index

DVT formation was observed until POD 21 in 95 patients (27.2%). No thrombus was observed in 253 patients (72.8%). The DVT-positive ratio was 9.4% (6/64) in men and 31.3% (89/284) in women. There was a significant difference between the groups (*p* = 0.0002). The mean age in the DVT-positive group was 73.0 ± 7.9 years (range, 55-84 years), while that in the DVT-negative group was 63.3 ± 11.2 years (range, 39-90 years). Patients in the DVT-positive group were significantly older than those in the DVT-negative group (*p* = 0.0041).

We did not observe any patients with symptomatic PE. The mean BMI in the DVT-positive group was 22.4 ± 2.2 kg/m^2^ (range, 18.0-26.2 kg/m^2^), while that in the DVT-negative group was 22.9 ± 2.9 kg/m^2^ (range, 17.6-24.9 kg/m^2^), with no significant difference (*p* = 0.2956). The mean operating time was 135.1 ± 41.0 min (range, 93-154 min) in the DVT-positive group and 125.9 ± 20.03 min (range, 104-171 min) in the DVT-negative group, with no significant difference (*p* = 0.4509). In our hospital, all patients were administered enoxaparin at 2,000 units twice a day for two weeks after surgery and an intermittent pneumatic compression device during the perioperative period for thromboprophylaxis. The thrombus was diagnosed as the distal type in 89 patients (93.7% of the DVT-positive group). Thrombus occurred in the soleal vein in 71 patients (74.7% of the DVT-positive group), but there was no significant difference between the operated and non-operated sides. The thrombus in the popliteal vein (proximal type) was observed in 6 patients (6.3% of the DVT-positive group) (Table 3).

**Table 3:** Characteristics of patients in the DVT-positive group

	DVT+	DVT-
No. of patients (total 348)	95(27.2%)	253(72.8%)
Male (64)	6(9.4%)	58(90.6%)
Female (284) (P=0.0002)	89(31.3%)	195(68.7%)
Age(years) （P=0.041）	73.0±7.9(55-84)	63.3±11.2(39-90)
Operation time (min) （P=0.4509）	135.1±41.0(93-154)	125.9±20.3(104-171)
BMI (%) （P=0.2956）	22.4±2.2(18.0-26.2)	22.9±2.9(17.6-24.7)
Deep Venous Thrombosis		
Proximal	6(6.3%)	-
Distal	89(93.7%)	-
Symptomatic PE	0	-
Side of VTE		-
Operated side	39(41.0%)	-
Non-operated side	44(46.1%)	-
Both	12(12.8%)	-

BMI, body mass index; DVT, deep venous thrombosis; PE, pulmonary embolism, Proximal= iliac-popliteal vein, Distal=lower leg vein

**Figure 1:**
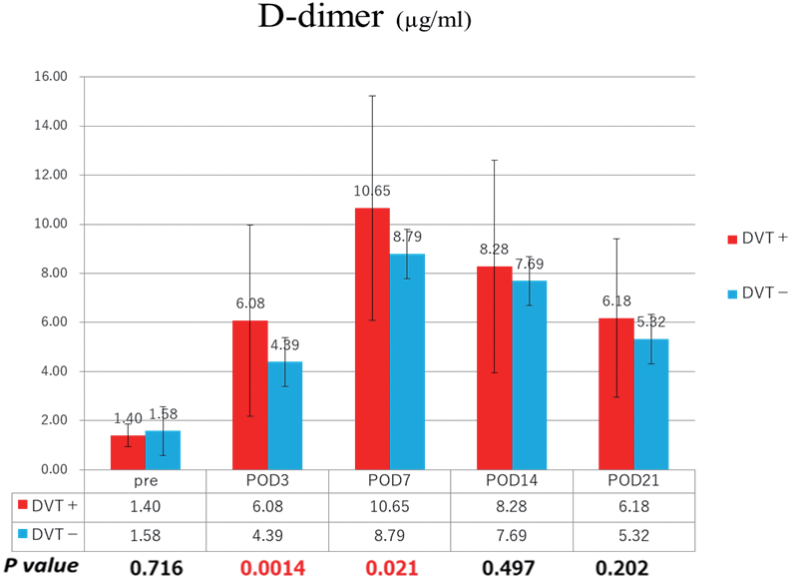
Changes in D-dimer values On POD 3 and POD 7, the D-dimer value was significantly higher in the DVT-positive group compared with the DVT-negative group (POD 3: 6.08 µg/ml in positive group vs. 4.39 µg/ml in negative group, P=0.0014; POD 7: 10.65 µg/ml in positive group vs. 8.79 µg/ml in negative group, P=0.021). POD, post-operative day; DVT, deep venous thrombosis

Postoperative changes in D-dimer values are shown in Figure 1. At POD 3 and 7, the D-dimer value was significantly higher in the DVT-positive group compared with the DVT-negative group (POD 3: 6.08 µg/ml in the positive group vs. 4.39 µg/ml in the negative group, p = 0.0014; POD 7: 10.65 µg/ml in the positive group vs. 8.79 µg/ml in the negative group, *p* = 0.021).

**Figure 2:**
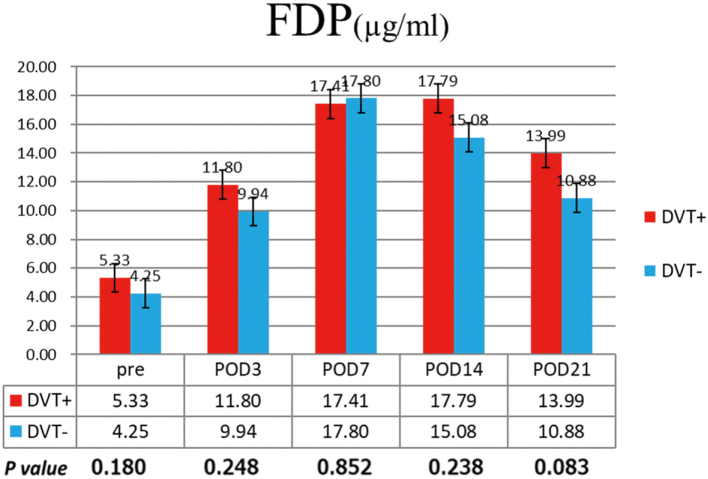
Changes in FDP values There was no significant difference in FDP values between the DVT-positive group and the DVT-negative group. DVT, deep venous thrombosis; FDP, fibrinogen degradation products

The postoperative changes in FDP values are shown in Figure 2. There was no significant difference in the FDP values between the DVT-positive and negative groups.

**Figure 3:**
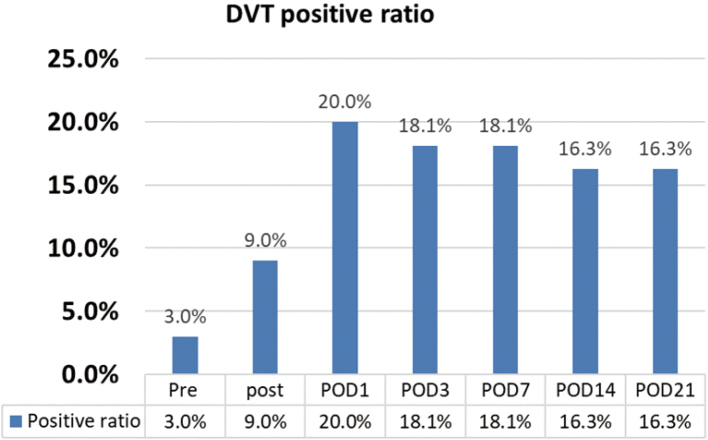
DVT-positive rates The DVT-positive rates in all cases were 3.0% pre-operatively, 9.0% on POD 0, 20.0% on POD 1, 18.1% on POD 3, 18.1% on POD 7, 16.3% on POD 14, and 16.3% on POD 21. POD, post-operative day; DVT, deep venous thrombosis

The changes in DVT-positive rates are shown in Figure 3. The DVT-positive rates were 3.0% preoperatively, 9.0% on POD 0, 20.0% on POD 1, 18.1% on POD 3, 18.1% on POD 7, 16.3% on POD 14, and 16.3% on POD 21.

**Figure 4:**
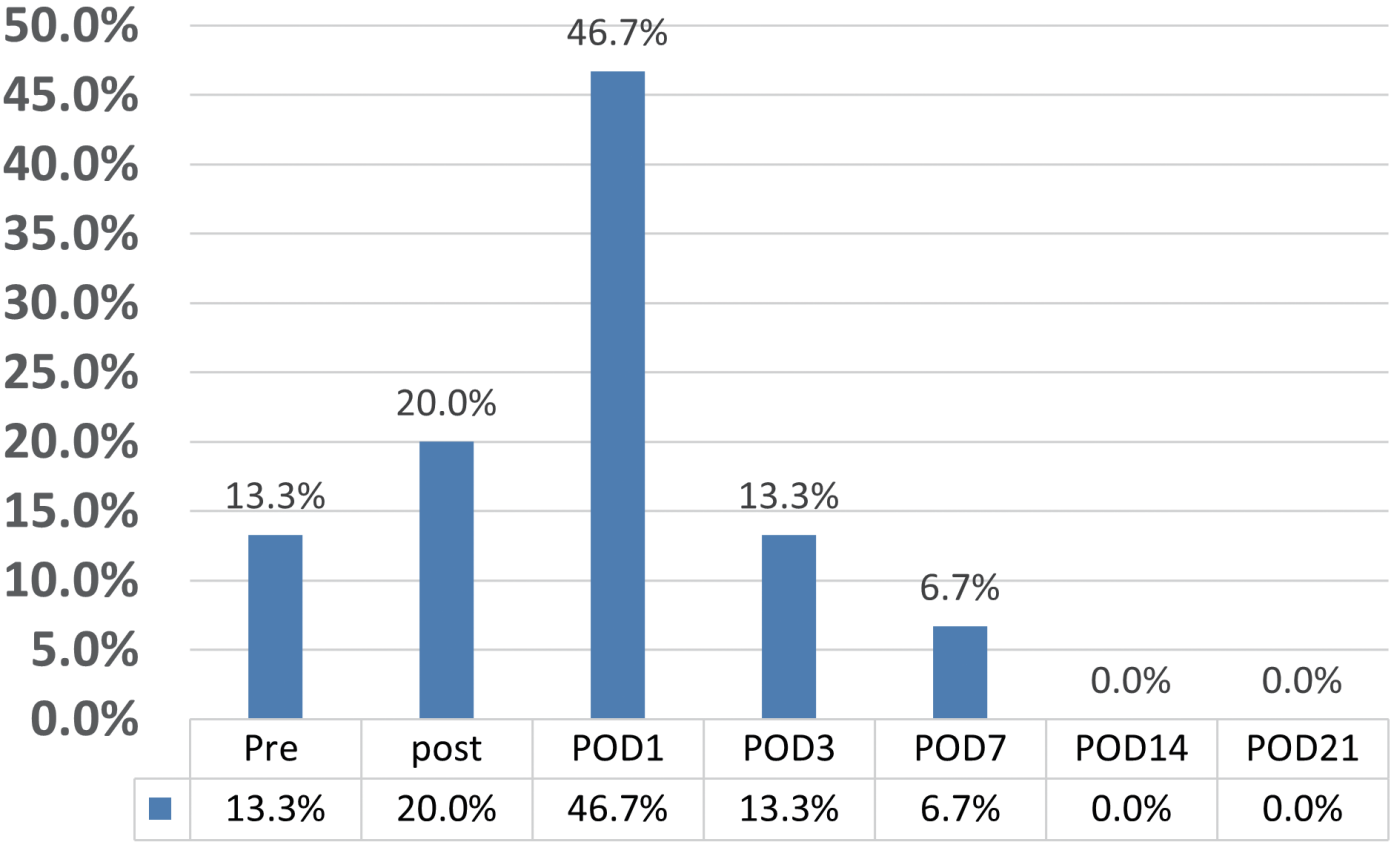
Time points when thrombus became positive for the first time in the DVT-positive group In the DVT-positive group, the frequencies for first observation of DVT were 20.0% on POD 0, 46.7% on POD 1, 13.3% on POD 3, 6.7% on POD 7, and 0% on POD 14 and POD 21. POD, post-operative day; DVT, deep venous thrombosis

Figure 4 shows the time points at which DVT was first observed. In the DVT-positive group, the frequencies for the first observation of DVT were 20.0% on POD 0, 46.7% on POD 1, 13.3% on POD 3, 6.7% on POD 7, and 0% on POD 14 and 21.

## Discussion

THA is a surgical procedure associated with a high risk of DVT. Therefore, physical and drug prophylaxis is recommended for THA performance. In our hospital, all patients were administered enoxaparin for thromboprophylaxis at 2,000 units twice a day for two weeks after surgery. Despite thromboprophylaxis, we observed DVT in 27.2% of the cases. This incidence of DVT was less than the rates of 34-63% in previous reports^3-9)^. In this study, thrombus mostly formed in the soleal vein, consistent with previous reports^10, 11)^.

In previous studies, the majority of thrombus cases were found in the lower leg and disappeared within a few days, but approximately 30% extended to the proximal side within a few weeks^12-14)^. Thrombus that develops on the proximal side tends to become free-floating within a short period, leading to widespread pulmonary thromboembolism^15)^. Therefore, the source of embolization for severe PE is often proximal to the popliteal vein, particularly the femoral vein. However, it can sometimes occur on the distal side^11, 15, 16)^, and from the soleal veins^11)^. Therefore, for distal thrombosis with a tendency towards enlargement, it is necessary to check for thrombus at regular intervals.

Regarding imaging diagnosis, noninvasive venous US is the first choice for thrombosis detection in the lower extremities because of high diagnostic accuracy^17-19)^. Contrast-enhanced CT examination involves administration of a contrast agent and radiation exposure. We sometimes encountered rare cases in which clinical VTE was highly suspected without positive US findings. In such cases, contrast-enhanced CT or magnetic resonance imaging should be performed^18, 20-22)^.

US can continuously visualize the femoral, popliteal, and lower leg veins in B mode. A compression US technique was used, in which the vein was compressed with a probe, allowing the determination of the presence or absence of a thrombus. Color, power, and pulse Doppler methods are commonly used to visualize blood vessels and check for obstructions^18, 23, 24)^. The mean sensitivity for proximal-type DVT in the femoral and popliteal veins, compared with venography, was reported to be 97% (range, 89-100%) for symptomatic patients and 62% (range, 38-100%) for asymptomatic patients^25)^. Furthermore, the mean sensitivity of US for distal-type DVT was 73% (range, 0-100%) for symptomatic patients and 53% (range, 0-92%) for asymptomatic patients^25)^. However, the specificity compared with venography was 96% (95% CI, 95.2-96.8%)^18)^. The result of 27% of DVT positive cases in our study may be lower than the actual result because the sensitivity of US is low in the distal-type. However, since the specificity is high, there is little possibility that false positives are included.

In the present study, the patients in the DVT-positive group were significantly older than those in the DVT-negative group. The risk of DVT was reported to gradually increase every 10 years with age, and the risk of DVT in patients aged >65 years was 2.1 times higher^26, 27)^. The results relative to the mean age in the present study correspond to those of previous studies. In addition, the DVT-positive ratio was 9.4% (6/64) in men and 31.3% (89/284) in women. There was a significant difference between the groups (*p* = 0.0002). The factors that cause DVT more frequently in women than in men may be that the average age of women is older than that of men and that older people are more likely to delay rehabilitation progress^28, 29)^. In addition, it is possible that lower preoperative activities of daily living due to advanced age may affect postoperative walking ability^28, 29)^, and lower limb pumping ability due to differences in muscle mass may contribute to venous stasis^30)^.

In the DVT-positive group, thrombus formation was observed in 66.6% of cases by POD 1, indicating that the thrombus appeared very early after surgery. This finding may be reflected by the high D-dimer value observed during the early postoperative period. Furthermore, body posture during surgery and bed rest until POD 1 may facilitate thrombus formation. However, there was no significant difference in the operation time between the DVT-positive and DVT-negative groups. In addition, there were no significant differences between the operated and non-operated sides. The affected femur and lower leg were always grasped by the assistant, and the limb position was continuously moved according to the surgical situation. Therefore, it is possible that the massage effect was equivalent to that on the healthy side to which the intermittent pneumatic compression device was attached. It is also possible that postoperative rehabilitation may be affected by the early start of range of motion training on the affected side. Regarding the operation, the duration of vein occlusion in the affected hip joint with flexion and the internal rotation position at the time of implant insertion may be intraoperative factors. However, we did not examine these factors in this study. Nevertheless, we can conclude that a distal thrombus formed very early after the operation. Moreover, we can clarify that many thrombus cases after THA were found by POD 1, and almost all thrombus formation was completed by POD 7.

Attention should be paid to PE because its results are serious. Nakamura et al.^31)^ investigated 108 acute PE patients and reported that 57% developed symptoms while standing or walking and 22% developed symptoms after defecation or urination. Therefore, we need to pay careful attention to the physical condition of patients when they get out of bed, depending on the tendency for increased thrombus formation. In high-risk patients, it is necessary to check for thrombus formation using US to prevent symptomatic PE preoperatively and when they get out of bed. Therefore, as we did, it is better to perform blood sampling and a lower limb US test during the perioperative period and to perform contrast-enhanced CT for positive individuals. In this study, the previously mentioned prevention did not cause symptomatic PE in all subjects except the high-risk group.

A limitation of this study is that US was performed by one person, and hence, the inter-rater reliability cannot be calculated accurately.

In conclusion, the majority of thrombus cases were found on POD 1, and thrombus formation was mostly completed by POD 7. The thrombus formed very early after the surgery, indicating that US should be performed as soon as possible during the perioperative period. In patients who have a tendency for thrombus formation postoperatively, it may be necessary to examine thrombus formation.

## Funding

No funding was received.

## Author contributions

MN, SK, YS, HO, SK obtained informed consent from the patients and performed total hip arthroplasty and various tests for research. MI supervised this study and provided advice and guidance.All authors read and approved the final manuscript.

## Conflicts of interest statement

Authors declare that there are no conflicts of interest.
